# Toward High Selectivity Aniline Synthesis Catalysis at Elevated Temperatures

**DOI:** 10.1021/acs.iecr.1c03695

**Published:** 2021-12-02

**Authors:** Clément G.A. Morisse, Annelouise M. McCullagh, James W. Campbell, Colin How, Donald A. MacLaren, Robert H. Carr, Chris J. Mitchell, David Lennon

**Affiliations:** † School of Chemistry, Joseph Black Building, University of Glasgow, Glasgow G12 8QQ, U.K.; ‡ School of Physics and Astronomy, Kelvin Building, University of Glasgow, Glasgow G12 8QQ, U.K.; § Huntsman Polyurethanes, Everslaan 45, 3078 Everberg, Belgium; ∥ SABIC UK Petrochemicals Ltd., The Wilton Centre, Redcar TS10 4RF, U.K.

## Abstract

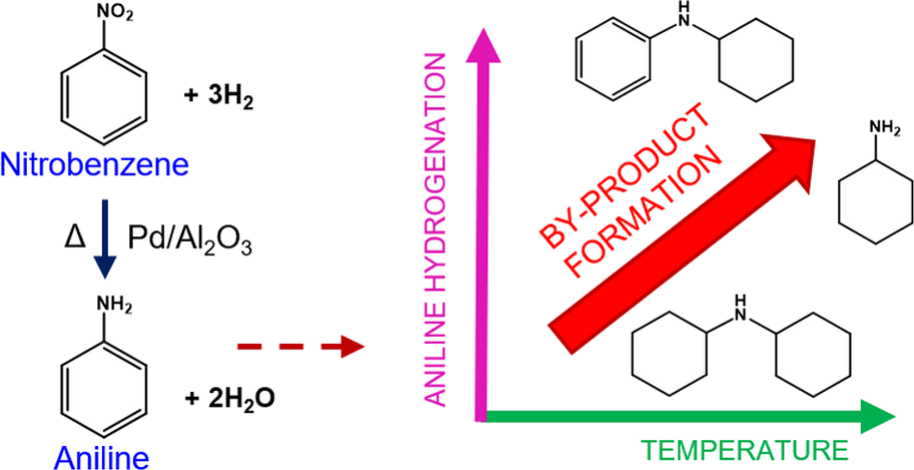

In connection with an initiative to enhance heat recovery from the large-scale operation of a heterogeneously catalyzed nitrobenzene hydrogenation process to produce aniline, it is necessary to operate the process at elevated temperatures (>100 °C), a condition that can compromise aniline selectivity. Alumina-supported palladium catalysts are selected as candidate materials that can provide sustained aniline yields at elevated temperatures. Two Pd/Al_2_O_3_ catalysts are examined that possess comparable mean Pd particle sizes (∼5 nm) for different Pd loading: 5 wt % Pd/Al_2_O_3_ and 0.3 wt % Pd/Al_2_O_3_. The higher Pd loading sample represents a reference catalyst for which the Pd crystallite morphology has previously been established. The lower Pd loading technical catalyst more closely corresponds to industrial specifications. The morphology of the Pd crystallites of the 0.3 wt % Pd/Al_2_O_3_ sample is explored by means of temperature-programmed infrared spectroscopy of chemisorbed CO. Reaction testing over the range of 60–180 °C shows effectively complete nitrobenzene conversion for both catalysts but with distinction in their selectivity profiles. The low loading catalyst is favored as it maximizes aniline selectivity and avoids the formation of overhydrogenated products. A plot of aniline yield as a function of WHSV for the 0.3 wt % Pd/Al_2_O_3_ catalyst at 100 °C yields a “volcano” like curve, indicating aniline selectivity to be sensitive to residence time. These observations are brought together to provide an indication of an aniline synthesis catalyst specification suited to a unit operation equipped for enhanced heat transfer.

## 1 Introduction

**Scheme 1:**
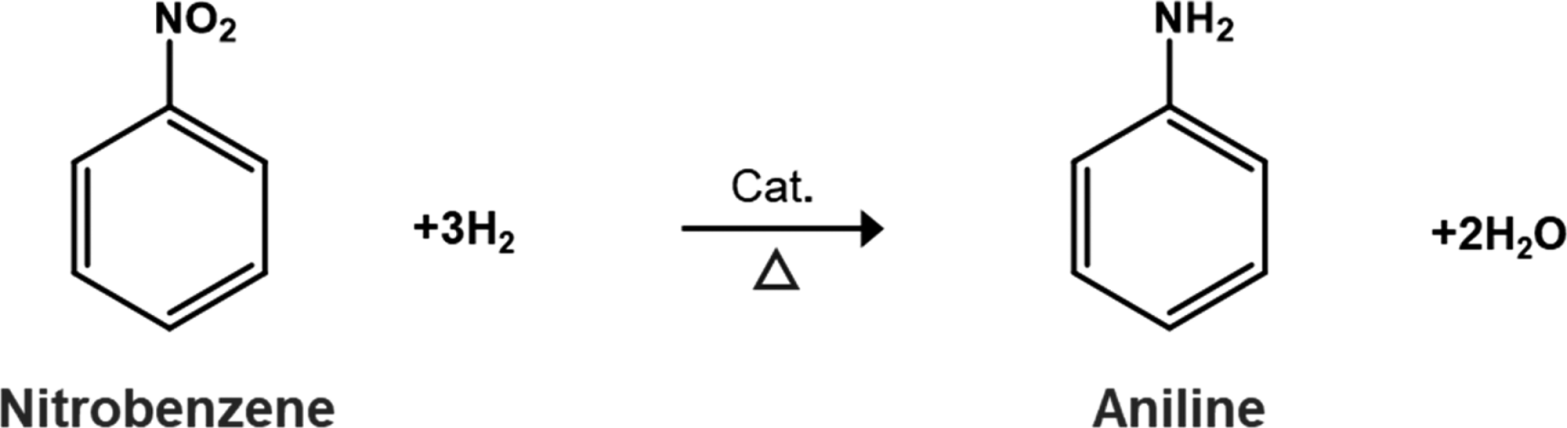
Hydrogenation of Nitrobenzene to Produce Aniline

Polyurethanes are classed as specialty polymers that find wide application in modern society. For example, they are used in fields as diverse as furnishings, the construction sector, insulation, and as components used in aircrafts and cars. Polyurethanes are prepared by the reaction between isocyanates and polyols. More than 90% of polyurethanes are produced from aromatic poly isocyanates, with toluene diisocyanate (TDI) and methylene diphenyl diisocyanate (MDI) being the dominant materials.^1^ Over recent years, the polyurethane industry has exhibited annual growth rates of 4–5%, with annual consumption in 2017 reported to exceed 20 Mt. This growth is expected to continue for the foreseeable future.^1^ MDI is produced via the condensation of aniline with formaldehyde in the presence of an acid (e.g., HCl).^2^ Thus, aniline is a major component of any large-scale isocyanate production chain, where it is usually produced by the heterogeneously catalyzed hydrogenation of nitrobenzene (Scheme 1). Kahl and co-workers have reviewed the catalyst and process options applied to large-scale aniline production, with reactions typically performed at full nitrobenzene conversion.^3^ It is estimated that in 2008 approximately 3.0 Mt of aniline were consumed in the production of isocyanates, mainly MDI.^3^

Due to the complexity of the MDI manufacturing process, not the least due to the involvement of phosgene as a reagent, isocyanate production is normally undertaken within an integrated chemical complex.^4^ In recent years, there has been an increasing intention to minimize energy costs and to improve overall operational efficiency of such facilities. One route to achieve these goals is to ensure efficient heat recovery from highly exothermic reactions.

The formation of steam in heat recovery processes is common^5−8^ and is achieved via the vaporization of water at 100 °C for standard pressures. Heating of steam exceeding its boiling point for a given pressure results in the formation of “dry steam”, or as it is otherwise known, superheated steam.^9^ Superheated steam is higher in energy than that of saturated steam; an energy increase of 30 kJ kg^–1^ is reported when comparing saturated steam (100 °C, 1 barg) to superheated steam (110 °C).^10^ This highlights the considerable energy output which may be harnessed by operating reactors at elevated temperatures to produce superheated steam which subsequently can be utilized for further chemical processes or, alternatively, to generate electricity.^11^ Within the context of an isocyanate production facility, the hydrogenation of nitrobenzene, which exhibits standard reaction enthalpies of −554.1 and −468.2 kJ mol^–1^ for the liquid and vapor phases, respectively,^12^ is ideally suited to raise superheated steam. However, this would require running the reaction at elevated temperatures (≥100 °C), a condition that can compromise catalytic selectivity.

Nitrobenzene hydrogenation with high aniline selectivity is achievable with a plethora of different metal-supported catalysts, including palladium,^13,14^ nickel,^15^ platinum,^16^ and copper.^17^ Pd-based catalysts exhibit high activity and preferential reduction of functional groups in proximity to aromatic systems and so are widely reported in literature for nitrobenzene hydrogenation at high aniline selectivity.^18−20^ Pd supported on alumina is suited to the higher temperature operational platform connected with heat recovery options relevant to large-scale aniline production facilities.

**Scheme 2:**
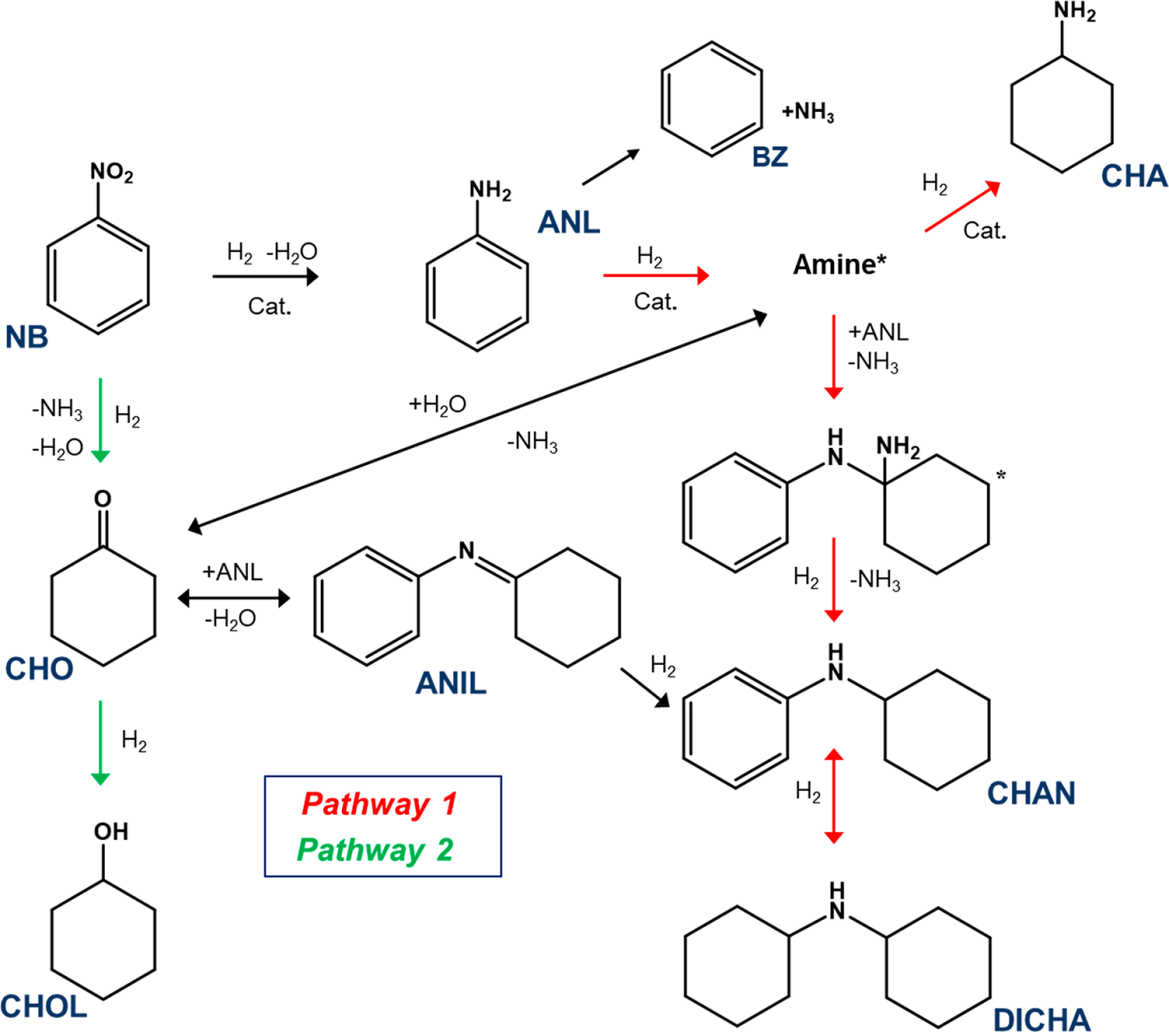
Reaction Scheme for Nitrobenzene (NB) Hydrogenation to Aniline (ANL) Pathway 1 [products: cyclohexylamine (CHA), *N*-cyclohexylaniline (CHAN), dicyclohexylamine (DICHA)] is highlighted in red, while pathway 2 [products: cyclohexanone (CHO) and cyclohexanol (CHOL)] is highlighted in green. Adapted with permission from ref (21). Copyright 2015 Wiley-VCH.

Sá Couto et al. have reported on outcomes linked to liquid phase nitrobenzene hydrogenation over a series of Pd/Al_2_O_3_ catalysts. Specifically, a parametric investigation of nitrobenzene hydrogenation with a 1 wt % Pd/Al_2_O_3_ catalyst in a 3-phase basket reactor revealed increasing temperature as the parameter which primarily influenced the production of secondary byproducts, more so than was observed for increasing pressure (14–30 barg) or initial nitrobenzene concentration (3–10 wt % NB).^21^ Benzene was isolated as a byproduct of nitrobenzene hydrogenation and proposed to emerge from hydro-denitrogenation of aniline^21^ (Scheme 2). It is noted however that the scheme presented by Sá Couto et al. does not address the formation of azobenzene, azoxybenzene, or hydrazobenzene as observed by, for example, Gelder et al.^14^

Clearly, Scheme 2 shows the hydrogenation of nitrobenzene over Pd/Al_2_O_3_ to be a more complicated process than that implied by Scheme 1, with several pathways accessible that can compromise aniline selectivity. The first pathway, and most prominent, occurs from direct overhydrogenation of aniline resulting in the production of cyclohexylamine (CHA), *N*-cyclohexylaniline (CHAN), and dicyclohexylamine (DICHA) (Scheme 2, red), with DICHA being the final hydrogenation product.^21^ A second pathway (Scheme 2, green) is associated with the direct transformation of nitrobenzene to cyclohexanone (CHO), which can then be further hydrogenated to cyclohexanol (CHOL). Further work by Sá Couto et al. considered the effect of varying operational parameters on catalytic activity for different Pd loadings and particle diameters.^22^ More recently, Sá Couto et al. reported an increase in aniline selectivity with increasing time on stream using a 0.3 wt % Pd/Al_2_O_3_ catalyst in a trickle bed reactor, which is attributed to the blocking of active sites via carbon deposition that hinders production of heavy byproducts (ANIL {*N*-cyclohexylideneaniline}, CHAN, DICHA).^23^

The matter of morphological effects of supported Pd catalysts applied to selective hydrogenation reactions is a well-established concept.^24−26^ This article builds on the previous studies but works toward more precisely defining the surface chemistry connected with sustained aniline production. Specifically, byproduct formation of a particular catalyst formulation needs to be definitively established to guide the selection of a suitable postreaction purification stage (i.e., distillation unit) for any intended plant revisions at the industrial complex.^27,28^ To this end, two Pd/Al_2_O_3_ catalysts are examined: a 5 wt % Pd/Al_2_O_3_ catalyst and a 0.3 wt % Pd/Al_2_O_3_ technical-grade catalyst. The former represents a reference catalyst, where the morphology of the Pd crystallites has been previously examined by diffuse reflectance infrared Fourier transform spectroscopy (DRIFTS).^29^ In contrast, the lower loading sample is potentially more suited to industrial application on cost grounds; its catalytic performance and morphological attributes will be compared to the reference material. Although a reduced Pd loading has economic benefits, the lower metal loading may result in a mass-transfer limited regime.^30,31^ To overcome this issue, the 0.3 wt % Pd/Al_2_O_3_ catalyst has been prepared to possess an “egg-shell” distribution of Pd, where a thin shell of Pd is concentrated near the edge of the catalyst pellets.^31,32^ Importantly, the two catalysts exhibit comparable Pd particle sizes, thereby enabling particle size independent morphology deductions to be made that can be directly linked to a propensity for byproduct formation. The article is constructed as follows. Both catalysts are comprehensively characterized, with the morphology of the low loading sample evaluated by application of CO chemisorption coupled with temperature-programmed infrared spectroscopy, a common method for determination of adsorption sites.^33^ IR measurements of chemisorbed CO on low metal loading catalysts presents significant sensitivity issues arising from the reduced Pd content, thus the development of a method to permit collection of decipherable and meaningful IR spectra for chemisorbed CO on the 0.3 wt % Pd/Al_2_O_3_ catalyst is described. Reaction testing is performed in the vapor phase to facilitate postreaction analysis. Reaction profiles over the temperature range of 60–180 °C performed in the presence of excess hydrogen show both samples to display high nitrobenzene conversions but distinct selectivity profiles. Concentrating on the lower Pd loading sample, aniline yield is shown to be sensitive to residence time. The results are discussed in terms of Pd morphology and operating conditions that minimize overhydrogenation reactions. Overall, the study makes a connection between byproduct formation and catalyst specification that includes an awareness of Pd crystallite morphology.

## 2 Experimental Section

Two catalysts were used in this study: a 5 wt % Pd/Al_2_O_3_ sample (powder) obtained from Alfa Aesar (ref: 11713) and a 0.3 wt % Pd/Al_2_O_3_ technical egg-shell catalyst (pellets) that was supplied by Huntsman Polyurethanes (ref: ASC-1). Henceforth, the 5 wt % catalyst will be referred to as GU-1 and the 0.3 wt % catalyst as GU-2.

### 2.1 Catalyst Characterization

Palladium loading was measured by atomic absorption spectrophotometry (AAS) by means of a PerkinElmer Analyst 100 instrument (λ = 244.8 nm) that was calibrated from a 1 g L^–1^ Pd/HCl commercial stock solution (Sigma-Aldrich). Samples were prepared for analysis by dissolving the catalyst sample (0.1 g) in aqua regia and boiling for 30 min with fumes allowed to evaporate. After cooling, deionized water (5 mL) was added and the solution filtered into a 25 mL volumetric flask prior to measurement. Brunner-Emmett-Teller (BET) total surface area measurements were carried out on a Micromeritics ASAP 2400 gas adsorption analyzer using a static barometric method. Catalyst samples (0.5 g) were placed into a glass tube and outgassed at 140 °C overnight in flowing nitrogen. Adsorption of nitrogen was completed at −196 °C. Surface areas were calculated using the BET method.^34^ CO adsorption isotherms obtained at 298 K using a pulse-flow method utilizing an in-line gas chromatograph (Thermo Finnigan, Trace GC, TCD detector) were used to determine the chemisorption capacity of both catalysts. With the assumption of a surface stoichiometry of CO:Pd = 1:2,^35^ these values were used to estimate Pd mean particle size. Transmission electron microscopy (TEM) was performed on a Tecnai T20 microscope with an accelerating voltage of 200 keV. Samples were prepared by dispersing the powder catalysts in methanol. The suspension was then dropped on a micron scale carbon grid (300 μm mesh grid, Agar scientific) and dried in a vacuum desiccator. Particle size analysis was performed with ImageJ software using the particle size routine applied to an ensemble of particles from a number of images collected from representative areas of the sample. Powder X-ray diffraction (XRD) was performed with a Siemens D5000 diffractometer (source accelerating voltage: 40 kV; source intensity: 40 mA) using Cu Kα (1.5418 Å) radiation in Bragg-Brento geometry (range: 5–80° θ). XRD patterns were monitored using a scan rate of 0.02 deg s^–1^.

### 2.2 Temperature-Programmed Infrared Spectroscopy

*In situ* infrared experiments were performed with a Nicolet Nexus FTIR spectrometer fitted with a SpectraTech Smart diffuse reflectance cell and environmental chamber. GU-1 was supplied as a powder and was used directly. However, in order to obtain reasonable signal/noise IR spectra for CO chemisorption on the low loading catalyst (GU-2), it was necessary to employ a sample handling stage. Namely, scrapings of the outer layer of the catalyst pellets (diameter ca. 1 mm) were taken using a scalpel and placed in the sample cup of the IR cell. Isolating the outer layer of egg-shell catalysts using razors has previously been applied in TEM measurements to enhance nanoparticle numbers and permit ease of measurement.^36,37^ Thereafter, treatment of the samples was comparable for both catalysts. Reduction of the Pd nanoparticles was undertaken in a flow of He (BOC gases, 99.9%) and H_2_ (BOC gases, 99.8%) while heating to 110 °C and held at this temperature for 30 min. The temperature was then increased to 200 °C for 1 h, with H_2_ flow stopped after 30 min and the sample allowed to return to ambient temperature in flowing He, where a background IR spectrum was acquired. The sample was exposed to CO (CK gases, 99.99%) and subsequently flushed with He to remove nonchemisorbed CO from the environmental chamber. Spectra were recorded at 28 °C (520 scans at 4 cm^–1^ resolution). For desorption experiments, the catalyst was heated *in situ* under He flow and maintained at the targeted temperature for 10 min before cooling to 28 °C for spectral acquisition. This process was repeated for 50, 100, 150, 200, 250, 300, 350, 400, and 450 °C for the 5 wt % Pd/Al_2_O_3_ sample (GU-1). CO desorption temperatures up to 200 °C were explored for GU-2. Due to the low metal content of this sample, the S/N ratio of the spectra were inferior to those of the higher loading sample, such that desorption measurements exceeding 200 °C were uninformative. Spectra are presented as difference spectra, where the spectrum of a clean, activated catalyst has been subtracted from that of a CO-dosed spectrum. No additional spectral treatment was performed.

### 2.3 Nitrobenzene Hydrogenation

Reaction testing was carried out in the vapor phase using a plug flow reactor (1/4” Swagelok, internal diameter: 0.18”) arrangement housed in a split tube furnace (LPC Elements). H_2_ (25 mL min^–1^) and He (12.5 mL min^–1^) were supplied by mass flow controllers (Brooks, 5850 TR series). Nitrobenzene was supplied as a vapor using a heated bubbler system that delivered 0.028 μmol (nitrobenzene) s^–1^. The nitrobenzene was premixed with hydrogen to give a H_2_:nitrobenzene molar flow ratio of ca. 600:1. This large hydrogen excess was selected to expose the accessibility of all hydrogenation pathways illustrated in Scheme 2. With the consideration of scale-up options, it is important to establish what byproducts could be formed when using these Pd/Al_2_O_3_ catalysts. GU-1 was used as received, GU-2 pellets were crushed and sieved to give a powder with particle size of 500–250 μm. Activation of catalysts (mass ∼27 mg) utilized a flow of He/H_2_ (35/15 mL min^–1^) and a temperature ramp (5 °C min^–1^) up to 200 °C. The temperature was held for 1 h with H_2_ flow stopped after 30 min. All gas lines leading to and exiting from the reactor were kept at a fixed temperature (60 °C) using heating tape (Electrothermal, HT95508) to ensure compounds were retained in the vapor phase. Analysis was carried out using gas–liquid chromatography via an Agilent 6850 series II fitted with a Durabond DB-17 capillary column (30 m, 0.250 mm, 0.5 μm) and an FID detector. GLC samples were taken using a 250 μL gas-sampling valve. Reaction components were identified with respect to retention times of standards and quantified using response factors derived from individual calibration curves. A catalyst conditioning phase, in which the reaction was run at 60 °C for 16 h, was utilized to allow the reaction to stabilize prior to data collection. Replicate data points were collected under steady-state conditions for the following temperatures: 60, 80, 100, 120, 140, 160, and 180 °C, with data presented as an average value.

Nitrobenzene conversion was calculated according to eq 1,^38^
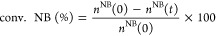
1where *n*^NB^(0) represents the initial number of moles of nitrobenzene and *n*^NB^(*t*) the number of moles of nitrobenzene at time *t*. Product selectivity values were calculated according to eq 2,^38^
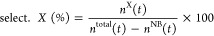
2where *n*^X^(*t*) represents the number of moles of compound *X* at time *t* and *n*^total^(*t*) the total number of moles of all observed compounds at time *t.* Aniline yield values were calculated according to eq 3,^38^
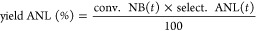
3where conv. NB(*t*) represents percent nitrobenzene conversion at time *t* and select. ANL(*t*) the percent selectivity of aniline at time *t*. Turnover frequencies (TOF), defined as molecules reacting per second per surface metal atom,^39^ were calculated according to eq 4,
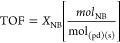
4where *X*_NB_ is a fraction representing nitrobenzene conversion for a given reaction, *mol*_NB_ represents the total number of moles of nitrobenzene per second and *mol*_Pd(s)_ represents the number of moles of surface Pd available.

### 2.4 Reaction Profiles As a Function of Weight Hourly Space Velocity

Experiments assessing how the reaction profile for GU-2 was influenced by residence time were undertaken by varying the weight hourly space velocity (WHSV) at a reaction temperature of 100 °C and an incident nitrobenzene flow of 0.034 μmol s^–1^. Catalyst masses of ca. 25, 60, 100, 200, and 500 mg were used that, respectively, corresponded to WHSV values of 0.65, 0.29, 0.15, 0.08, and 0.03 h^–1^. Catalyst activation and nitrobenzene hydrogenation reaction conditions, excluding reaction temperature, remained as described above.

## 3 Results and Discussion

### 3.1 Catalyst Characterization

#### 3.1.1 AAS, BET, and CO Chemisorption and TEM

Table 1 summarizes the catalyst characterization measurements. Palladium loadings of 4.3 ± 0.3 and 0.31 ± 0.03 wt % were observed for GU-1 and GU-2, respectively. Total surface areas for GU-1 and GU-2 were 140 ± 10 and 120 ± 8.6 m^2^ g^–1^. Powder XRD patterns are presented in Figure S1 and indicate the two catalysts to be based around different support materials. Whereas the reference catalyst (GU-1) used exclusively γ-alumina, the support material for the technical catalyst (GU-2) contained mixed δ and θ phases. Saturation CO values for GU-1 and GU-2 were 51.0 and 3.85 μmol g_(cat)_^–1^, respectively. However, on normalization with respect to the number of Pd atoms present, their dispersion values and, indeed, calculated mean Pd particle size are comparable (GU-1 = 4.3 ± 0.08 nm, GU-2 = 4.0 ± 0.06 nm, and mean particle size = 4.2 nm).

**Table 1:** Surface Area, Uptake of CO, Metal Dispersion, Particle Size, and Concentration of Surface Pd Atoms for GU-1 and GU-2

catalyst	nominal loading (wt %)	actual Pd loading (AAS) (wt %)	BET (m^2^ g^–1^)	saturation coverage of CO (μmol CO g^–1^_(cat)_)	Surface Pd atoms (μmol g_(cat)_^–1^)	catalyst dispersion^b^ (%)	calc. mean Pd particle size (nm)	observed mean Pd particle size (TEM) (nm)
**GU-1**	5	4.3	140 ± 10.0	51 ± 0.96	102	24.2	4.3 ± 0.08	5.0 ± 0.88^a^
**GU-2**	0.3	0.31	120 ± 8.6	3.85 ± 0.06	7.69	27.2	4.0 ± 0.06	*ca.* 5.0^c^

a TEM measured GU-1 mean Pd particle size was derived from 87 Pd crystallites.

b Catalyst dispersion values are calculated from CO uptake.

c Only 3 Pd crystallites were identified via TEM due to the low metal loading and poor contrast observed for this sample.

**Figure 1:**
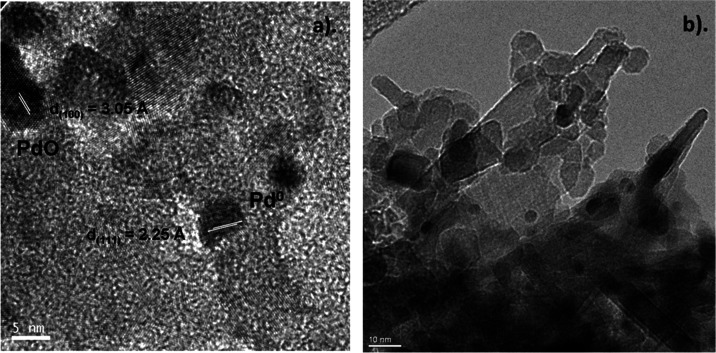
TEM micrographs: (a) GU-1 (scale bar = 5 nm) and (b) GU-2 (scale bar = 10 nm). The abundance of particles from the GU-1 sample permitted an estimate of crystallite *d*-spacing.

Representative transmission electron micrographs are presented in Figure 1; contrast within the images derives from both atomic number and diffractive effects, the latter making it harder to discriminate small nanoparticles from a crystalline support. Within Figure 1a, it is possible to discern lattice spacings of the crystallites, which signify the presence of PdO (*d*_(100)_ = 3.05 Å) and Pd metal (*d*_(111)_ = 2.25 Å) dispersed within an amorphous alumina matrix. Figure S2 presents the particle size distribution for GU-1, which is centered about 5 nm. The low Pd loading of GU-2, and weaker contrast between the metal and support (which appears more crystalline than that of GU-1), complicated a statistically significant determination of Pd particle size for this sample, but the Pd particles were estimated to be *ca*. 5 nm in diameter. The TEM-derived mean Pd particle sizes are consistent with those obtained from CO chemisorption (Table 1).

#### 3.1.2 CO Temperature-Programmed IR Spectroscopy

**Figure 2:**
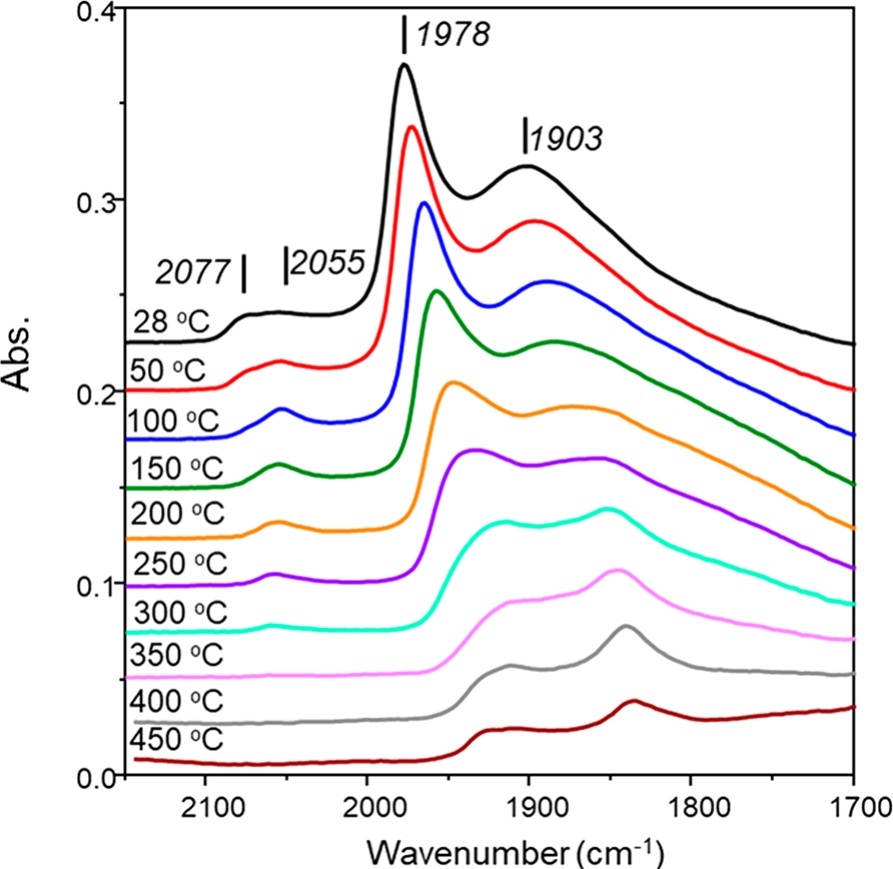
CO temperature-programmed IR spectra for GU-1 (28–450 °C). The spectra have been offset by 0.025 au to facilitate viewing.

The temperature-programmed IR spectroscopic profile for CO chemisorption over GU-1 has recently been reported over the temperature range of 28–300 °C.^29^Figure 2 extends this range to 450 °C. The room temperature spectrum depicts 4 features: a broad band at 1903 cm^–1^ assigned to μ_3_ bridge-bonded CO on Pd(111) planes, a sharp band at 1978 cm^–1^ arising from μ_2_ bridge-bonded CO on Pd(100) planes, and a broad feature at higher wavenumbers that can be resolved to a band at 2055 cm^–1^ that is associated with linear CO adsorption to edge sites and a shoulder feature at 2077 cm^–1^ arising from linear CO adsorption to corner sites.^40^ The significance of distinguishing between CO adsorption on corner and edge sites, adsorption sites proposed to be involved in hydrogen supply,^40−42^ via DRIFTS has previously been considered.^29^ Furthermore, and central to this study, is to note that the profile of the ambient temperature infrared spectrum shown in Figure 2 is indicative of the Pd crystallites adopting a truncated cuboctahedron structure.^40^ Thus, catalytic turnover on GU-1 can be considered as occurring on the Pd surface sites, as revealed within Figure 2.

Figure 2 shows linearly adsorbed CO to fully desorb from GU-1 in the ranges of 50–100 °C and 300–350 °C for corner and edge sites, respectively, indicating that the edge atoms represent high energy sites. Bridge-bonded CO was held more strongly on the catalyst and was still present in the spectrum collected after the maximum desorption temperature of 450 °C. These results agree with previous CO TP-IR measurements for 5 wt % Pd/Al_2_O_3_ catalysts,^29,40^ indicating GU-1 to provide a useful reference material on which to consider morphological effects on hydrogenation activity.

**Figure 3:**
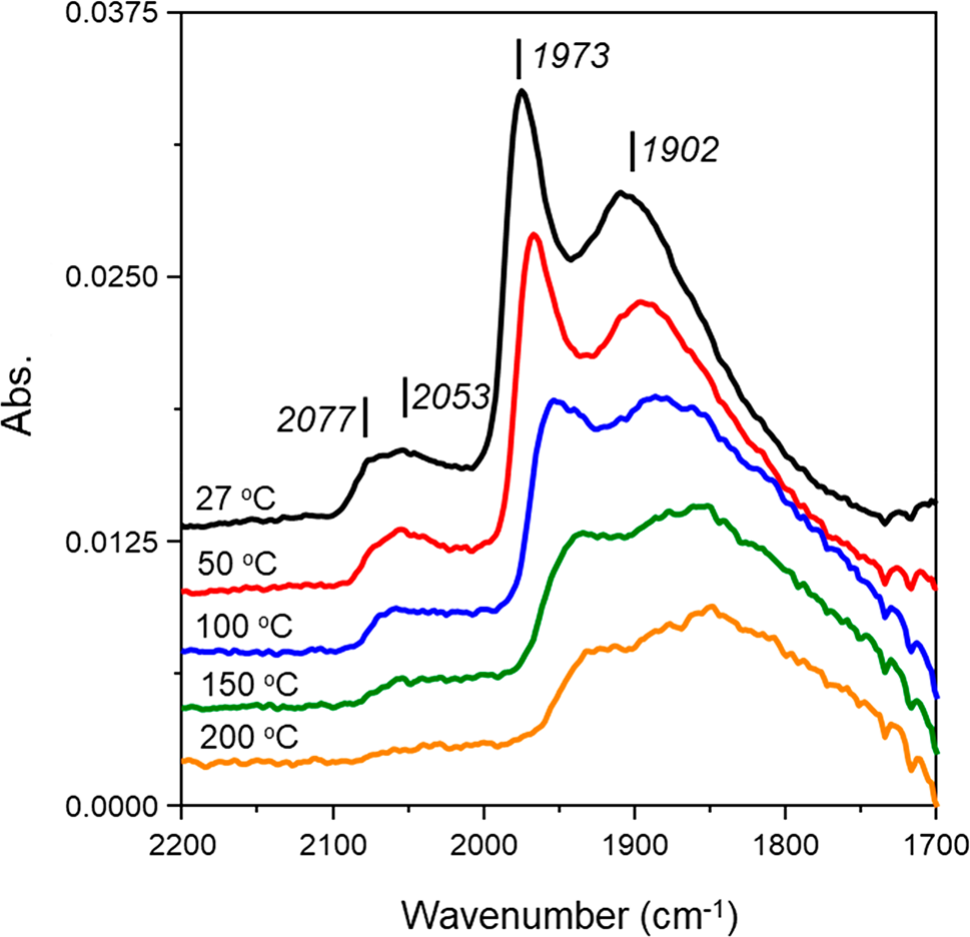
CO temperature-programmed IR spectra for GU-2. Note spectra have been offset by 0.025 au to facilitate viewing.

Figure 3 gives the CO TP-IR spectra for GU-2 recorded over the temperature range 27–200 °C. As considered in Section 2.2, due to the relatively low density of surface sites [7.7 μmol Pd_(s)_ g_(cat)_^–1^*cf*. 102 μmol Pd_(s)_ g_(cat)_^–1^], desorption temperatures ≥ 200 °C led to insufficient CO_(ad)_ to produce measurable spectra. Accepting the lower concentration of Pd in GU-2, the room temperature spectrum of Figure 3 corresponds closely to that of GU-1, with peaks observed at 2077, 2053, 1973, and 1902 cm^–1^.

The strength of CO adsorption on GU-2 follows the trend observed with GU-1: bridge-bonded CO > linear (edge) CO > linear (corner) CO. CO adsorbed linearly to corner sites desorbed in the range of 50–100 °C and bridge-bonded CO (μ_2_ and μ_3_) was present at the maximum desorption temperature of 200 °C. In contrast, CO adsorbed linearly on edge sites appears to desorb in the range of 150–200 °C, a significant reduction compared to GU-1 (300–350 °C). It is possible that this difference in spectral profiles merely reflects the lower spectral intensity encountered with the lower loading sample. Importantly, on comparing Figure 3 with Figure 2, GU-2 is seen to possess a similar profile to that encountered with GU-1, indicating the morphology of the particles of the industrial grade, low Pd loading variant to be comparable to the reference catalyst. Moreover, as Section 3.1.1 shows both samples to possess comparable mean Pd particle size (∼5 nm), GU-1 can be used to infer insight on the morphological trends connected with hydrogenation activity of the industrial specification Pd/Al_2_O_3_ catalyst (GU-2).

### 3.2 Reaction Testing

**Figure 4:**
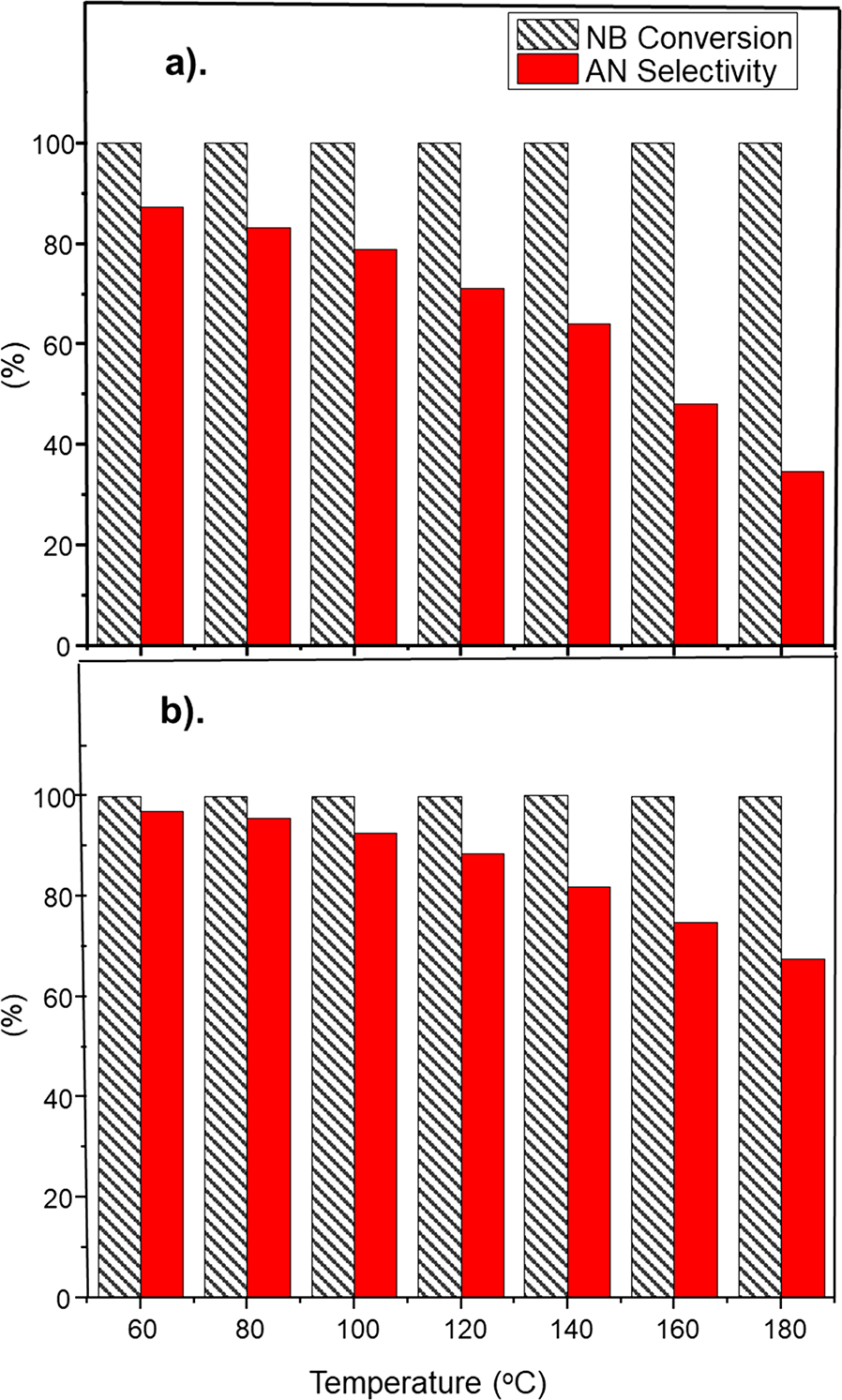
Nitrobenzene conversion (gray hatched columns) and aniline selectivity (red columns) as a function of temperature recorded at a WHSV of 0.46 h^–1^: (a) GU-1 and (b) GU-2. Temperature ramping was initiated after a 16 h reaction conditioning phase undertaken at 60 °C.

Figure 4 shows that near complete nitrobenzene conversion was achieved with both catalysts throughout the temperature range studied: GU-1 ≥ 99.92% and GU-2 ≥ 99.96%. This was facilitated by the elevated temperatures and large excesses of hydrogen used (H_2_:NB = ca. 600:1). Each catalyst data set corresponds to a different turnover frequency (TOF): TOF_(GU-1)_ = 0.01 s^–1^ and TOF_(GU-2)_ = 0.13 s^–1^. TOF is determined with respect to the nitrobenzene conversion, the number of moles of nitrobenzene, and the number of moles of surface Pd.^39^ As nitrobenzene flow was fixed and nitrobenzene conversion was near complete for both data sets, variances in TOF reflect the different metal loadings associated with GU-1 and GU-2. Operation at full nitrobenzene conversion is representative of the industrial scenario.^3^ Comparison of aniline selectivity for both data sets (Figure 4) showed a decrease with increasing reaction temperature for both GU-1 and GU-2; however, the exact aniline selectivity values varied. A maximum of 88% aniline selectivity was observed for GU-1, which decreased to 35% as reaction temperatures were elevated to 180 °C. In contrast, GU-2 exhibited an initial aniline selectivity of 97% at 60 °C that decreased to 68% with increasing temperature. Thus, the lower Pd weighting catalyst (GU-2) shows greater aniline selectivity than GU-1 under the stated conditions. Blank reaction testing on a reference γ-alumina sample revealed minimal nitrobenzene conversion, therefore it is deduced that all the hydrogenation activity is attributed to the presence of the Pd nanoparticles for GU-1. The authors were unable to obtain a source of the mixed phase alumina associated with the technical catalyst (GU-2), therefore a probable role for the support in the chemistry facilitated over this material is unknown.

Consideration of the distribution of byproducts observed during hydrogenation is critical for determining variations in catalytic activity and for product purification on scale-up. Byproducts detected during testing of GU-1 and GU-2 revealed that the dominant cause for loss of aniline selectivity in both cases was owed to the overhydrogenation of aniline. Overhydrogenation of aniline during nitrobenzene hydrogenation is well-documented^21,22,43^ and is reported to arise via an amine intermediate that is either hydrogenated to give cyclohexylamine (CHA) or coupled with aniline to give a phenylamine intermediate, which then undergoes subsequent hydrogenation to N-cyclohexylaniline (CHAN) and finally dicyclohexylamine (DICHA).^21^

**Scheme 3:**
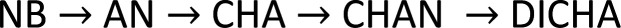
Proposed Stepwise Hydrogenation Pathway for Aniline Derived Byproducts.

**Figure 5:**
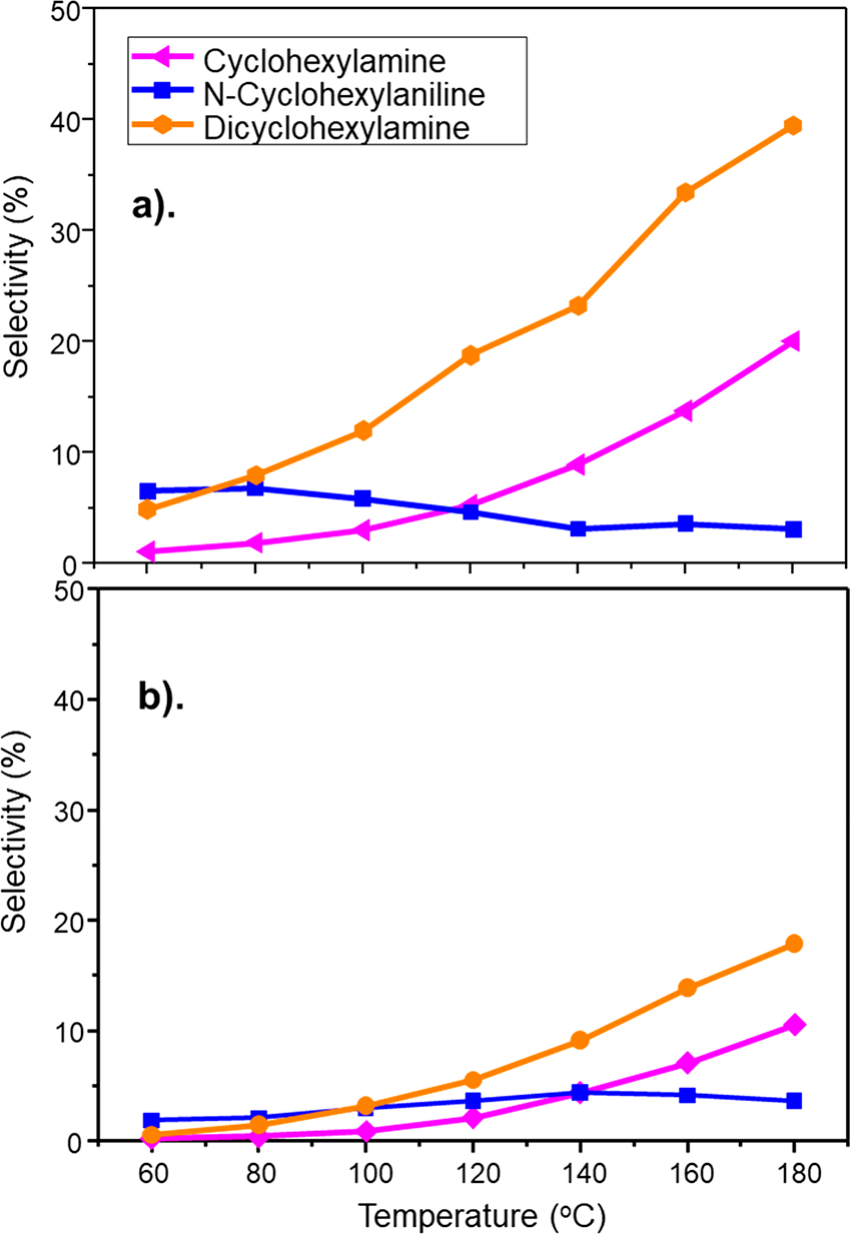
Selectivity of aniline overhydrogenation byproducts [pathway 1] CHA (pink), CHAN (blue), and DICHA (orange) as a function of increasing temperature at a WHSV of 0.46 h^–1^: (a) GU-1 and (b) GU-2. Temperature ramping was initiated after a 16 h reaction conditioning phase undertaken at 60 °C.

Selectivities to the byproducts derived from aniline hydrogenation are presented in Figure 5. Reflecting the trends observed in Figure 4, the extent of aniline hydrogenation byproduct formation is significantly greater for GU-1, but nevertheless, comparable trends are observed for both catalysts: namely, [DICHA] > [CHA] > [CHAN]. This reproducibility of trends for both catalysts is suggestive of a stepwise hydrogenation pathway as illustrated in Scheme 3.

Clearly, GU-1 facilitates aniline hydrogenation to a greater extent than observed for GU-2. As GU-1 and GU-2 have similar Pd particle sizes (Section 3.1.1) and distribution of Pd sites (Section 3.1.2), the loss of aniline selectivity associated with GU-1 compared to GU-2 can be assigned to the presence of a higher quantity of Pd crystallites and, importantly, not any structural difference between the two catalysts. Whereas Figure 5 shows DICHA to be the major product, this outcome differs from the liquid phase reaction results reported by Sá Couto and co-workers, who report higher levels of CHA and CHAN than DICHA.^21^ It is possible that this difference in product selectivity is attributable to differences in Pd crystallite morphology or, alternatively, reflects mass transfer issues associated with the liquid phase reaction.

**Figure 6:**
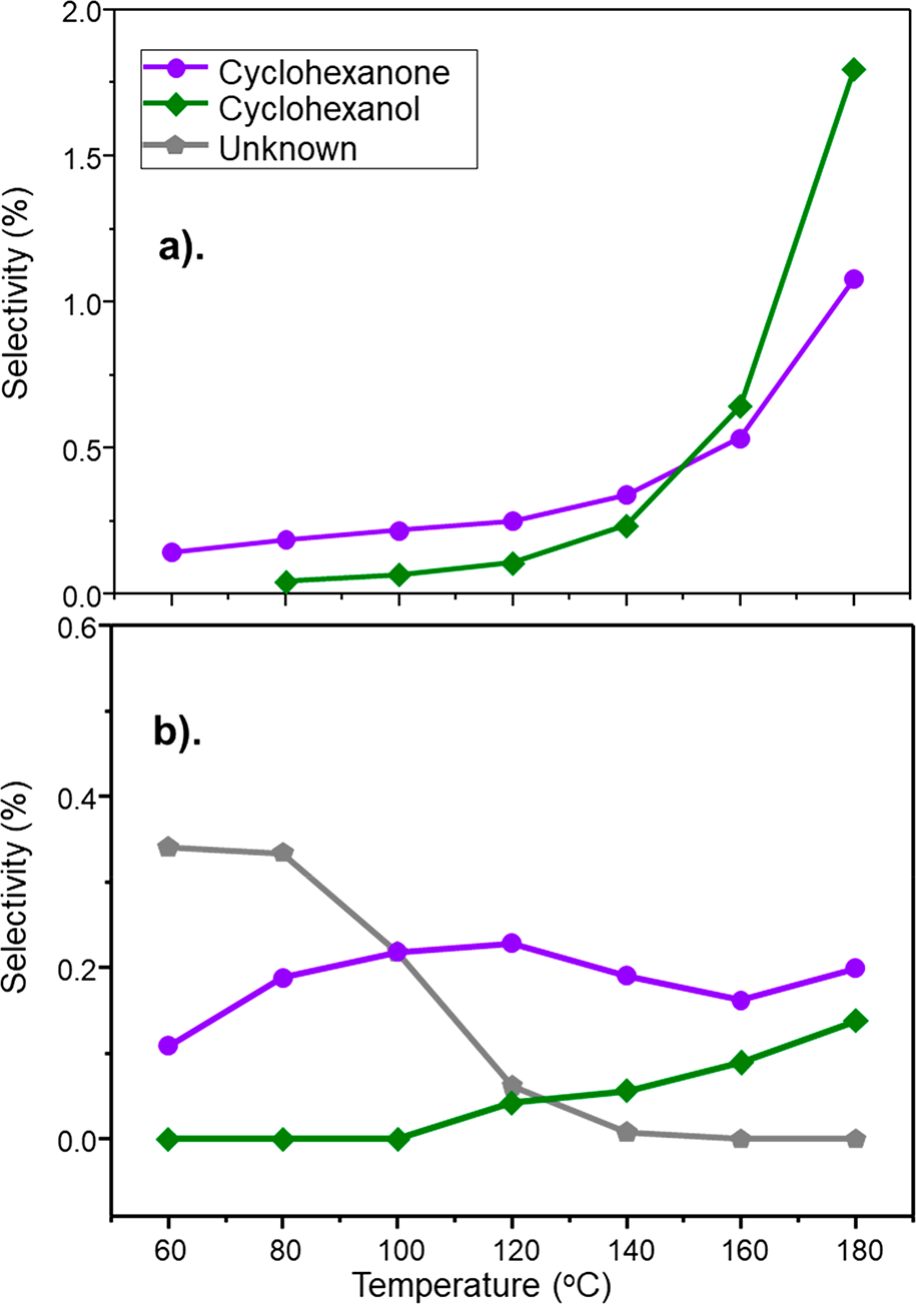
Selectivity of byproducts derived from nitrobenzene intermediates CHO (purple) and CHOL (green) as a function of increasing temperature at a WHSV of 0.46 h^–1^: (a) GU-1 and (b) GU-2. Temperature ramping was initiated after a 16 h reaction conditioning phase undertaken at 60 °C. An unknown byproduct was uniquely detected with GU-2. This moiety is indicated by gray pentagons, with the concentration estimated using the CHO GC response factor.

Figure 6 shows that further byproducts are observed, which are classified as nitrobenzene-derived intermediates, cyclohexanone (CHO) and cyclohexanol (CHOL), with CHOL being a hydrogenation product of CHO.^21^ Additionally, at temperatures < 160 °C, an unknown species is detected for GU-2 (Figure 6b). As its retention time is very close to that of CHO, it is thought to be an intermediate between a nitrobenzene-derived surface species and CHO. Work is underway to identify this molecule, whose GC retention time does not correspond to any obvious candidate. Products (CHO and CHOL) derived from this route are referred to as pathway 2 byproducts (Scheme 2, green).

The extent of pathway 2 byproducts is much less than that observed for pathway 1. A maximum selectivity for a pathway 1 derived byproduct of ca. 40% (DICHA, Figure 5a) was observed for GU-1. Comparatively, the maximum selectivity observed for pathway 2 derived byproducts for GU-1 was *ca*. 1.8% and owed to CHOL production (Figure 6a). This dramatic variation in byproduct distributions between the 2 pathways investigated here cements the nature of the Pd/Al_2_O_3_ catalysts to favor hydrogenation reactions over other transformations (e.g., coupling reactions involving nitrobenzene derived intermediates^21^) and highlights the relevance, and indeed requirement, of characterizing catalyst sites involved in hydrogen supply. As considered previously, Pd edge and corner sites are thought to play an important role in maintaining hydrogen supply.^29^ With reference to Figure 2 that shows edge sites to exhibit the higher enthalpy of adsorption of the terminal sites, it is tentatively suggested that these sites are active in facilitating dissociative adsorption of dihydrogen^29^ that, subsequently, participates in surface-mediated hydrogenation reactions.

A combination of Figures 4, 5, and 6 indicate that although the extent of byproduct formation is greater with GU-1, nevertheless, GU-2 displays comparable profiles involving the same chemical entities. This comparison demonstrates that comparable surface chemistry is observable in each case with Figure 3, indicating that GU-2 possesses a similar active site distribution to that observed for the reference catalyst GU-1 (Figure 2). Thus, it is the higher concentration of ∼5 nm Pd crystallites of GU-1 that is responsible for the greater degree of byproduct formation. Finally for this section, it is additionally noted that the difference in support materials for the two catalysts (Section 3.1.1) appears not to have unduly influenced the product distributions observed, confirming the surface chemistry evident in Figures 4, 5, and 6 to be Pd-mediated.

### 3.3 GU-2: Weight Hourly Space Velocity Dependence

Section 3.2 showed the technical catalyst GU-2 to exhibit superior aniline selectivity compared to the reference catalyst. Therefore, this catalyst was scrutinized further by examining reaction profiles at an elevated temperature as the weight hourly space velocity was varied in the range of 0.65–0.03 h^–1^. A reaction temperature of 100 °C was selected for these WHSV studies, which provide insight on whether extended contact with the catalyst can affect catalytic performance. Section 3.2 indicated overhydrogenation of aniline to be the primary cause of selectivity loss, thus reaction testing for WHSV focused on these downstream products (Scheme 2, pathway 1).

**Figure 7:**
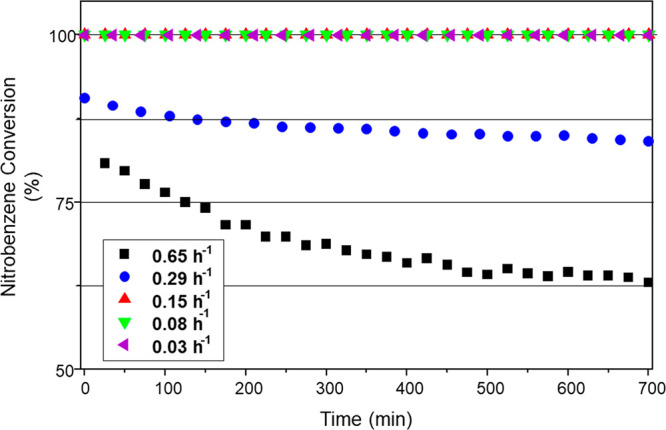
Nitrobenzene conversion for GU-2 as a function of time-on-stream and operation at different WHSV values: 0.65 (black), 0.29 (blue), 0.15 (red), 0.08 (green), and 0.03 (purple) h^–1^. Reaction temperature: 100 °C; feed ratio, H_2_:NB = 600:1; incident nitrobenzene flux = 0.034 μmol (nitrobenzene) s^–1^; and total gas flow = 37.5 mL min^–1^.

Figure 7 presents nitrobenzene conversion as a function of time-on-stream for 5 WHSV values: 0.65, 0.29, 0.15, 0.08, and 0.03 h^–1^. For WHSV values ≤ 0.15 h^–1^, complete nitrobenzene conversion was observed, whereas for WHSV values ≥ 0.29 h^–1^, only partial conversion was achieved. In the case of WHSV = 0.29 h^–1^, conversion was reasonably constant throughout the 700 min reaction period studied: initial nitrobenzene conversion = 89%; final conversion = 84%. In contrast, the WHSV of 0.65 h^–1^ showed a decline in nitrobenzene conversion from an initial value of 81% to a final value of 63%. Thus, it appears that a catalyst deactivation channel is evident on operation at higher space velocities. Figure 7 also indicates that a minimum residence time is required to ensure full nitrobenzene conversion. This is because the relatively low Pd loading of GU-2 means that short residence times with this material leads to insufficient contact with surface Pd atoms to effect full conversion. However, for a fixed feedstock flow rate, extending catalyst mass increases the exposure to Pd_(s)_, thereby increasing nitrobenzene consumption.

**Figure 8:**
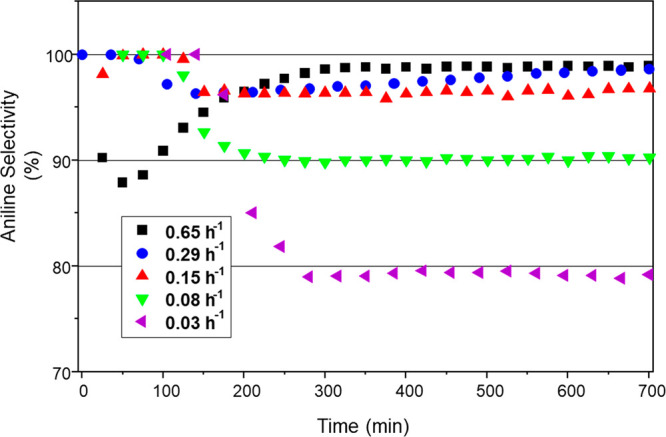
Aniline selectivity for GU-2 as a function of time-on-stream and operation at different WHSV values: 0.65 (black), 0.29 (blue), 0.15 (red), 0.08 (green), and 0.03 (purple) h^–1^. Reaction temperature: 100 °C; feed ratio, H_2_:NB = 600:1; incident nitrobenzene flux = 0.034 μmol (nitrobenzene) s^–1^; and total gas flow = 37.5 mL min^–1^.

Figure 8 shows the corresponding aniline selectivity values as a function of time-on-stream as WHSV values are varied and reveals some distinct trends. Selectivity for a WHSV of 0.65 h^–1^ showed an initial dip to ca. 87%; however, after ∼300 min run time, selectivity recovered and settled at 99%. This was the only WHSV value to show an increase in aniline selectivity with increasing run time. WHSV values of 0.29 and 0.15 h^–1^ exhibited similarly high aniline selectivities of ca. 98 and 97%, respectively, at steady-state. Significant declines in aniline selectivity were observed for WHSV’s of 0.08 and 0.03 h^–1^, with respective steady-state selectivities of 90 and 79% observed. This trend is indicative of a catalyst conditioning phase during the initial ∼100 min of the reaction. In this way, Figures 7 and 8 are indicating a degree of complexity within the reaction system, which the authors attribute to the dynamics of the hydrogenation process. Specifically, for a fixed incident hydrogen/nitrobenzene feed over GU-2, with an excess of hydrogen relative to hydrocarbon, catalyst masses equal to and exceeding 200 mg (WHSV: ≤0.08 h^–1^) signify the stage at which the surface hydrogen supply (via dissociative adsorption of dihydrogen) exceeds the nitrobenzene adsorption rate and, consequently, leads to the formation of higher hydrogenated products that compromise product selectivity. Thus, Figure 8 indicates that in the presence of a large hydrogen excess over a low loading technical grade catalyst that WHSV is an operational parameter. Equally, this analysis additionally signifies that incident hydrogen concentrations could also be adjusted to reduce the probability of the occurrence of overhydrogenation reactions.

**Figure 9:**
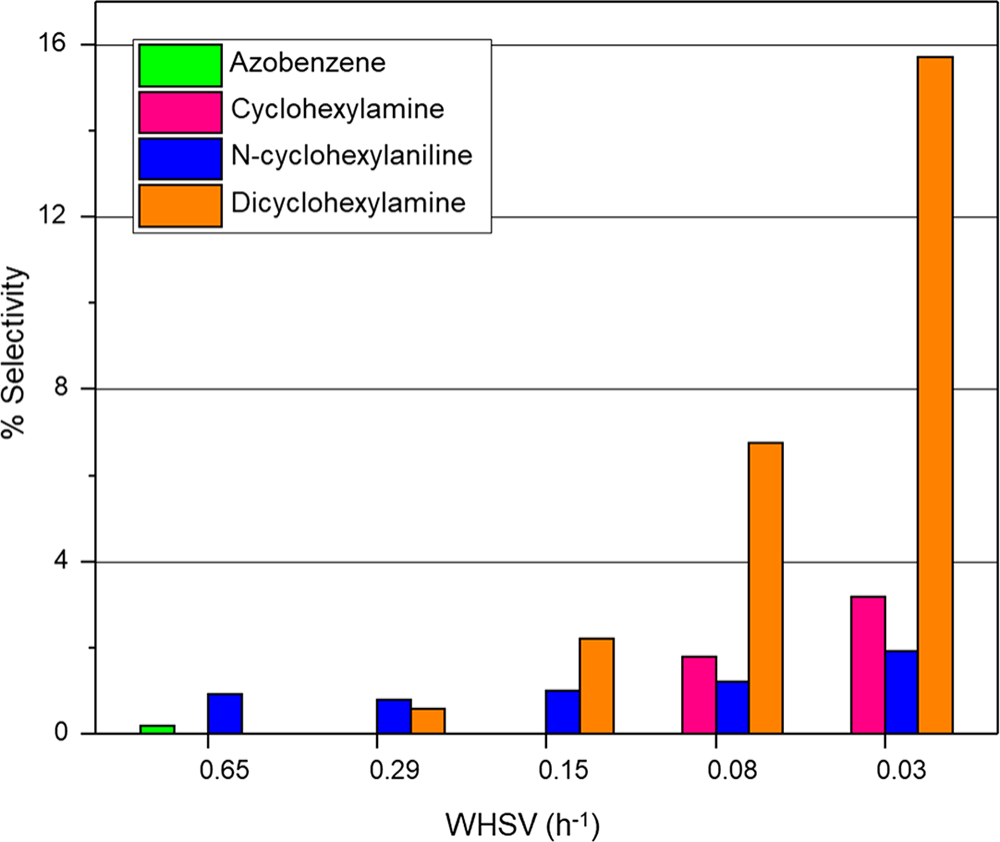
Plot of GU-2 byproduct selectivity at steady-state operation as a function of WHSV. Reaction temperature: 100 °C; feed ratio, H_2_:NB = 600:1; incident nitrobenzene flux = 0.034 μmol (nitrobenzene) s^–1^; and total gas flow = 37.5 mL min^–1^. Pathway 2 derived products CHO and CHOL (Scheme 2, green) are not included in this plot but were present in quantities < 0.2%.

Figure 9 complements Figure 8 by presenting a profile of how the byproducts are distributed as a function of WHSV. For the fixed (excess) hydrogen flow rate considered in this study, increased quantities of Pd in the reactor results in increased formation of aniline overhydrogenation byproducts. As the WHSV decreases (increasing catalyst mass), Figure 9 shows that the extent of DICHA formation increases. This observation is consistent with the proposal outlined in **Scheme 3**, in which DICHA is the final product in a consecutive hydrogenation pathway. It is interesting to note that the extent of CHAN formation appears to be insensitive to WHSV. This outcome may be rationalized with reference to Scheme 3, with CHAN being an intermediate between CHA and DICHA, and it is the interplay between the concentration of CHA and the rapid hydrogenation of CHAN to DICHA that sustains the CHAN concentration at an approximately constant level within a consecutive reaction sequence. Sá Couto et al. invoke a different route for CHA formation (Scheme 2).^21^ Work is underway to further evaluate the validity of Scheme 3.

At the highest WHSV of 0.65 h^–1^Figure 9 reveals the presence of a small quantity of azobenzene (AZO) alongside a larger component of CHAN. The AZO is thought to be derived from the coupling of nitrobenzene-derived intermediates nitrosobenzene and phenylhydroxylamine, which is indicative of an incomplete pathway in aniline formation.^13,14^ This was the only occasion when potentially hazardous azo compounds were encountered over the two catalysts studied. Figure 9 indicates such processes to be inherently disfavored over GU-2, although possible to a limited degree at high space velocities where hydrogenation pathways are less favored.

**Figure 10:**
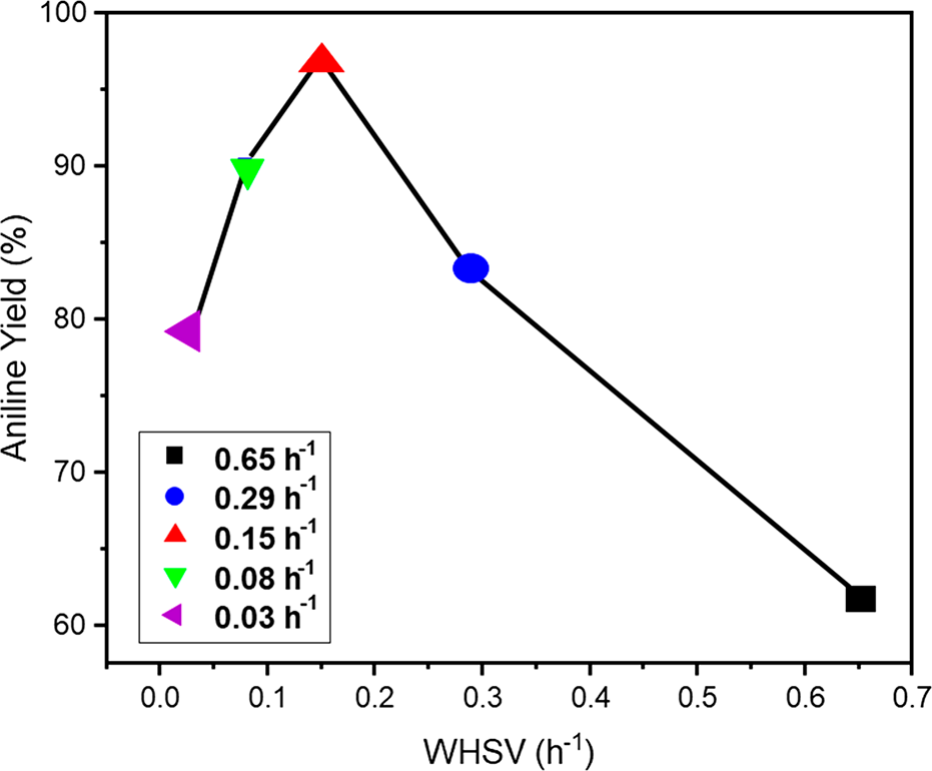
Plot of aniline yield as a function of increasing WHSV for GU-2. Reaction temperature: 100 °C; feed ratio, H_2_:NB = 600:1; incident nitrobenzene flux = 0.034 μmol (nitrobenzene) s^–1^; and total gas flow = 37.5 mL min^–1^.

Figure 10 provides a plot of aniline yield as a function of WHSV under steady-state conditions over GU-2 at 100 °C and shows that a WHSV of 0.15 h^–1^ (catalyst mass 100 mg) provides the optimal conditions for a maximized aniline yield of 97% within the stated configuration. This outcome is close to industrial performance specifications.^3^

Bringing these outcomes together, for a defined Pd crystallite morphology (as defined by Figures 2 and 3) operating within a fixed nitrobenzene feed rate and excess hydrogen, relatively high Pd loadings will lead to a loss of aniline selectivity due to overhydrogenation of predominantly aniline-derived products. As indicated in Figure 10, a matching of catalyst contact times can mitigate product loss for the low loading industrially relevant catalyst specification. The catalysts can be operated to avoid the formation of nitrobenzene-derived coupling products, which simplifies purification requirements for reactor exit streams. Future work will examine further how Pd particle morphology may be more explicitly linked to sustained aniline yields. A correlation between catalyst specification and byproduct formation during operation at elevated temperatures are prerequisite design inputs for energy efficient large-scale aniline synthesis plants.

## 4 Conclusions

Two Pd/Al_2_O_3_ catalysts (GU-1 and GU-2) have been examined for their suitability as aniline synthesis catalysts for application in a unit operation with suitable heat recovery capability. Nitrobenzene hydrogenation in the vapor phase has been investigated in a microreactor arrangement in the presence of a large hydrogen excess. These conditions were selected to expose the hydrogenation pathways accessible with these catalysts. The following conclusions can be drawn. (i) GU-1 and GU-2 possess comparable Pd particle sizes of ∼5 nm. Comparisons with GU-1 as a reference catalyst, combined CO chemisorption and TPIR spectroscopic measurements, indicate Pd nanoparticles of the low Pd loading technical catalyst to approximate to truncated cuboctahedra. (ii) Reaction testing over the temperature range of 60–180 °C shows that the smaller concentration of Pd crystallites of GU-2 significantly reduces overhydrogenation of aniline, a major cause for loss of product selectivity. (iii) Although the extent of byproduct formation is greater with GU-1, nevertheless, GU-2 displays comparable profiles involving the same chemical entities. This indicates that comparable surface chemistry is observable in each case and is consistent with the deduction that GU-2 possesses a similar active site distribution to that observed for the reference catalyst (GU-1). (iv) The presence of two major byproduct formation pathways is confirmed: pathway 1, the direct overhydrogenation of aniline; pathway 2, the transformation of nitrobenzene to cyclohexanone, which can then be further hydrogenated to cyclohexanol. Pathway 1 dominates and is thought to represent a consecutive hydrogen addition process. (v) For GU-2 at 100 °C and WHSV values ≤ 0.08 h^–1^, aniline selectivity is compromised via product over reduction. (vi) A plot of aniline yield versus WHSV for GU-2 at 100 °C yields a “volcano” type curve that peaks at an aniline selectivity of 97% at a WHSV value of 0.15 h^–1^. (vii) There is evidence of a catalyst conditioning period that affects aniline selectivity.

## Supporting Information Available

(Figure S1) XRD diffraction patterns for GU-1 and GU-2 and (Figure S2) TEM-derived particle size distribution of Pd crystallites for GU-1 (PDF)
