# Supplementary material for: Toward High Selectivity Aniline Synthesis Catalysis at Elevated Temperatures
Source: Ind Eng Chem Res. 2021 Dec 2;60(49):17917–27. doi: 10.1021/acs.iecr.1c03695 (PMC8802303; doi:10.1021/acs.iecr.1c03695)
Supplement: Supplementary file 1 — ie1c03695_si_001.pdf [file ie1c03695_si_001.pdf]

Supporting Information

Towards high selectivity aniline synthesis catalysis at elevated temperatures

Clément G.A. Morisse [1], Annelouise M. McCullagh [1], James W. Campbell [1], Colin How [2], Donald A. MacLaren [2], Robert H. Carr [3], Chris J. Mitchell [4] and David Lennon [1*]

1. School of Chemistry, Joseph Black Building, University of Glasgow, Glasgow, G12 8QQ, UK.
2. School of Physics and Astronomy, Kelvin Building, University of Glasgow, Glasgow, G12 8QQ, UK.
3. Huntsman Polyurethanes, Everslaan 45, 3078 Everberg, Belgium.
4. SABIC UK Petrochemicals Ltd., The Wilton Centre, Redcar, TS10 4RF, UK.

*Email: David.Lennon@glasgow.ac.uk
Telephone: +44-141-330-4372

Figure S1. XRD diffraction patterns for (a) GU-1 and (b) GU-2. The red lines for (a) GU-1 indicate 2θ values for γ-alumina. The red lines for (b) GU-2 indicate 2θ values for δ-alumina whilst the green lines indicate 2θ values for θ-alumina.

Figure S2. TEM derived particle size distribution of Pd crystallites for GU-1.

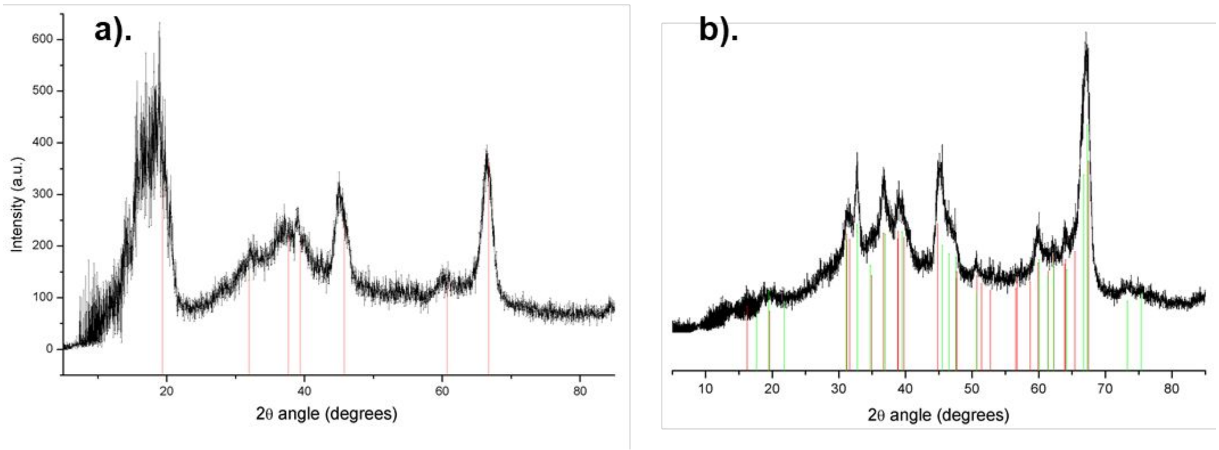

Figure S1. XRD diffraction patterns for (a) GU-1 and (b) GU-2. The red lines for (a) GU-1 indicate 2θ values for γ-alumina. The red lines for (b) GU-2 indicate 2θ values for δ-alumina whilst the green lines indicate 2θ values for θ-alumina.

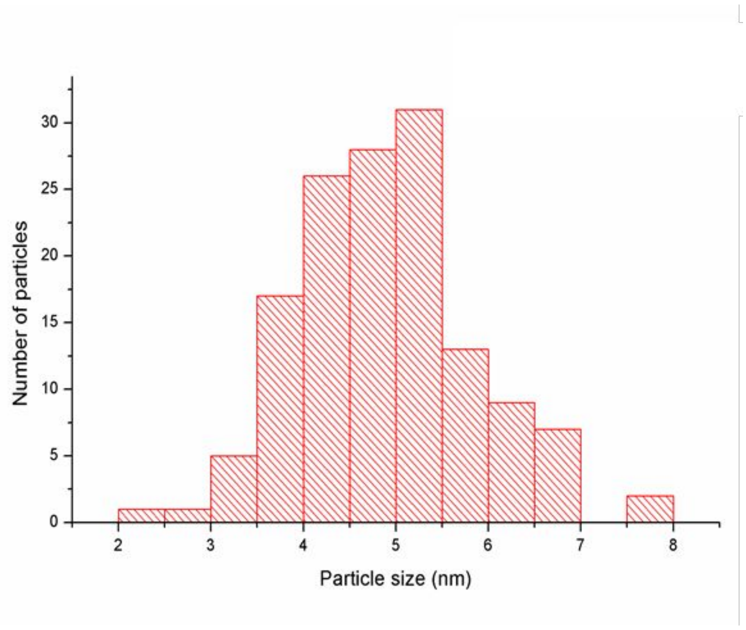

Figure S2. TEM derived particle size distribution of Pd crystallites for GU-1.
